# Thulium Fiber Laser Versus Holmium Laser for Ureteroscopic Lithotripsy: A Systematic Review and Meta-Analysis

**DOI:** 10.3390/medicina62040644

**Published:** 2026-03-28

**Authors:** Hyun Kyu Ahn, Jae Yong Jeong, Young Joon Moon, Dong Hyuk Kang, Hae Do Jung, Lawrence Kim, Kwang Hyun Kim, Joo Yong Lee

**Affiliations:** 1 Department of Urology, Ewha Womans University Seoul Hospital, Ewha Womans University College of Medicine, Seoul 07804, Republic of Korea; wharang11co@gmail.com (H.K.A.); jjunny74@naver.com (Y.J.M.); khkim.uro@gmail.com (K.H.K.); 2 Department of Urology, National Health Insurance Service Ilsan Hospital, Goyang 10444, Republic of Korea; urojjy@nhimc.or.kr; 3 Department of Urology, Inha University College of Medicine, Incheon 22332, Republic of Korea; dhkang@inha.ac.kr; 4 Department of Urology, Inje University Ilsan Paik Hospital, Inje University College of Medicine, Goyang 10380, Republic of Korea; haedojung@paik.ac.kr; 5 Department of Urology, Westmead Hospital, Westmead, NSW 2145, Australia; lawrence.kim@sydney.edu.au; 6 Faculty of Medicine and Health, The University of Sydney, Camperdown, NSW 2050, Australia; 7 Department of Urology, Severance Hospital, Urological Science Institute, Yonsei University College of Medicine, Seoul 03722, Republic of Korea; 8 Center of Evidence Based Medicine, Institute of Convergence Science, Yonsei University, Seoul 03722, Republic of Korea

**Keywords:** ureteroscopy, lithotripsy, laser, urolithiasis, thulium, Holmium, meta-analysis

## Abstract

*Background and Objectives:* This meta-analysis aimed to compare the clinical efficacy and safety of the Thulium fiber laser (TFL) and Holmium:Yttrium-Aluminum-Garnet (Ho:YAG) laser for ureteroscopic lithotripsy, considering the distinct technical characteristics of these two systems. *Materials and Methods:* Following the PRISMA guidelines and PROSPERO registration (CRD42023461573), a systematic search of PubMed, EMBASE, Cochrane Library, and Web of Science was conducted through August 2025. We included randomized controlled trials and non-randomized comparative studies comparing TFL and Ho:YAG laser in patients undergoing ureteroscopic management for urolithiasis. The primary outcomes were the stone-free rate (SFR) and complication rates (intraoperative and postoperative). *Results:* Thirteen studies involving 2217 patients were included. Overall, the TFL group demonstrated a significantly higher SFR compared to the Ho:YAG group (OR = 1.57, 95% CI 1.20–2.06, *p* = 0.001). In subgroup analysis, TFL showed superior SFR compared to Ho:YAG without pulse modulation (OR = 1.69, *p* = 0.01) and comparable efficacy to Ho:YAG with pulse modulation (OR = 1.52, *p* = 0.24). Regarding safety, no significant difference was observed in the intraoperative complication rate (OR = 0.77, 95% CI 0.35–1.70, *p* = 0.52) or the postoperative complication rate (OR = 1.02, 95% CI 0.65–1.60, *p* = 0.93) between the two groups. *Conclusions:* TFL provides a superior SFR compared to the Ho:YAG laser overall, a benefit primarily driven by its significant outperformance of standard Ho:YAG systems without pulse modulation. Importantly, TFL demonstrates comparable efficacy to modern Ho:YAG systems equipped with pulse modulation. The safety profile of TFL, including intraoperative and postoperative complications, is comparable to that of the Ho:YAG laser. Our findings suggest that TFL is a highly effective and safe modality for ureteroscopic lithotripsy, offering distinct advantages over standard Ho:YAG lasers while performing comparably to the latest pulse-modulated systems.

## 1. Introduction

Ureteroscopic lithotripsy has become the primary treatment modality for upper urinary tract urolithiasis. Current clinical guidelines, including those from the European Association of Urology (EAU) and the American Urological Association (AUA), advocate for ureteroscopy (URS) and retrograde intrarenal surgery (RIRS) as essential approaches for stone management [1,2,3]. The clinical applicability of these procedures has been significantly expanded by technological breakthroughs, such as the miniaturization of endoscopes and the development of high-performance optical fibers.

For decades, the Holmium:Yttrium-Aluminum-Garnet (Ho:YAG) laser has served as the gold standard for ureteroscopic lithotripsy [2,3]. However, traditional Ho:YAG systems have inherent limitations, including low wall-plug efficiency (1–2%) that necessitates bulky cooling systems, suboptimal wavelength matching for water absorption, and significant stone retropulsion [4].

To overcome these drawbacks, pulse modulation technologies (e.g., MOSES technology) have been introduced. These advancements optimize energy delivery to reduce retropulsion and improve fragmentation efficiency [5]. Despite these improvements, the fundamental physical constraints of the solid-state Ho:YAG medium remain. The recent emergence of the Thulium Fiber Laser (TFL) represents a potential paradigm shift in lithotripsy. TFL systems offer an electrical efficiency of approximately 12%, allowing for compact, air-cooled devices [6]. Crucially, the TFL emits a wavelength of 1940 nm, which aligns more closely with the water absorption peak (1940 nm) compared to the Ho:YAG laser (2100 nm). This physical property theoretically enables a four-fold-lower ablation threshold, facilitating efficient stone dusting [7]. Furthermore, TFL employs smaller-diameter silica fibers (50–150 μm), which occupy less space in the working channel, thereby enhancing irrigation flow and the deflection durability of flexible ureteroscopes [4].

While TFL possesses distinct theoretical advantages, it remains debated whether these physical properties translate into superior clinical outcomes compared to Ho:YAG lasers, particularly those equipped with modern pulse modulation. Previous clinical trials have reported inconsistent findings regarding stone-free rates and safety profiles. Therefore, we conducted this systematic review and meta-analysis to comprehensively compare the clinical efficacy and safety of TFL versus the Ho:YAG laser in ureteroscopic lithotripsy, with a specific focus on the impact of pulse modulation technology.

## 2. Materials and Methods

### 2.1. Inclusion Criteria

This systematic review and meta-analysis was conducted in accordance with the Preferred Reporting Items for Systematic Reviews and Meta-Analyses (PRISMA) guidelines (Table S1) and the Participants, Interventions, Comparators, Outcomes, and Study Design (PICOS) approach [8]. The inclusion criteria were: (a) patients with urolithiasis undergoing ureteroscopic lithotripsy (including semi-rigid URS and RIRS); (b) studies comparing TFL and Ho:YAG laser; and (c) reporting of clinical outcomes such as stone-free rate (SFR) or complication rates. Studies utilizing percutaneous nephrolithotomy (PCNL) or shock wave lithotripsy (SWL) were excluded to ensure the homogeneity of the surgical modality. We included randomized controlled trials (RCTs), non-randomized controlled trials (nRCTs), and prospective or retrospective cohort studies published as full-text peer-reviewed articles. Case reports, review articles, editorials, scientific congress abstracts, and studies without available full texts were strictly excluded. This study was exempt from institutional review board (IRB) approval as it synthesized previously published, de-identified data.

### 2.2. Search Strategy

A comprehensive search was performed in PubMed, EMBASE, the Cochrane Central Register of Controlled Trials (CENTRAL), and Web of Science for articles published up to August 2025. The search strategy utilized Medical Subject Headings (MeSH) and free-text terms including: (“thulium fiber laser” OR “TFL” OR “super-pulsed thulium fiber laser”) AND (“holmium:YAG” OR “Ho:YAG” OR “pulse modulation” OR “Moses technology”) AND (“urolithiasis” OR “calculi” OR “stone” OR “lithotripsy” OR “ureteroscopy” OR “URS” OR “retrograde intrarenal surgery” OR “RIRS”). Terms related to percutaneous nephrolithotomy (PCNL) were excluded to focus specifically on ureteroscopic management. Reference lists of relevant articles were manually screened to identify additional studies.

### 2.3. Study Selection and Extraction

Two researchers (D.Y.J. and H.K.A.) independently screened titles and abstracts, followed by full-text review. Extracted data included: primary author, publication year, country, study design, and patient characteristics. The primary outcome was SFR (defined as the absence of fragments or residual fragments < 4 mm on follow-up imaging). Secondary outcomes included intraoperative and postoperative complication rates (classified by the Clavien–Dindo system).

### 2.4. Quality Assessment

The Cochrane Risk of Bias (ROB) tool was used for RCTs, and the Methodological Index for Non-Randomized Studies (MINORS) was used for non-randomized studies. Evidence quality was further graded using the Scottish Intercollegiate Guidelines Network (SIGN) criteria. Disagreements were resolved through consensus or consultation with a third reviewer (J.Y.L.).

### 2.5. Statistical Analysis

The odds ratios (ORs) and 95% confidence intervals (CIs) were calculated for dichotomous variables, such as SFR and complication rates. For continuous variables, mean differences (MD) and 95% CIs were reported. Statistical heterogeneity among the included studies was evaluated using the chi-squared test, with a significance level of *p* < 0.05, and further quantified using the *I*^2^ statistic [9]. The Higgins *I*^2^ statistic was calculated as follows:

$$I^{2}=\frac{Q-df}{Q}\times 100\%$$

Considering the potential clinical heterogeneity among the included studies, a random-effects model was employed for all meta-analyses to provide a conservative estimate, regardless of the *I*^2^ value [10]. Meta-analysis was conducted using R software (version 4.3.1; R Foundation for Statistical Computing, Vienna, Austria) with the ‘meta’ package. Forest plots were generated to visually represent the pooled estimates and their respective CIs. To assess potential reporting bias, funnel plots were utilized, while L’Abbé plots and radial (Galbraith) plots were employed to further investigate the sources of heterogeneity [11,12]. Additionally, sensitivity analyses were performed to evaluate the robustness of our findings. Specifically, to address potential clinical heterogeneity arising from different definitions of surgical success, we conducted a sensitivity analysis stratifying the studies based on their definition of stone-free status (strict definition of 0 mm vs. relaxed definition of ≤2–4 mm residual fragments). Furthermore, subgroup analyses were conducted not only to compare TFL with Ho:YAG laser according to the presence or absence of pulse modulation technology, but also to stratify the outcomes based on stone location and procedural type (ureteral stones managed primarily with semi-rigid URS vs. renal or mixed stones managed with RIRS). This systematic review was prospectively registered with PROSPERO (CRD42023461573).

## 3. Results

### 3.1. Eligible Studies

**Figure 1:**
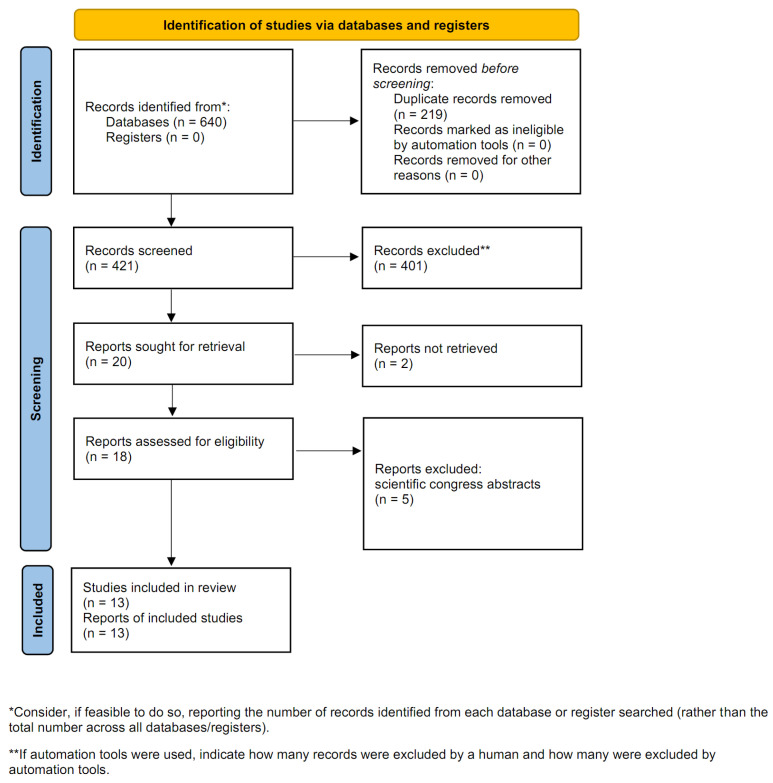
Study selection flow chart.

A total of 640 studies were initially identified through database searching. After removing 219 duplicates, 421 records remained for screening. Following a meticulous review of titles and abstracts, 401 studies were excluded. Specific reasons for exclusion included non-comparative study designs, use of surgical modalities other than ureteroscopy (e.g., PCNL), and publication types restricted to conference abstracts. Subsequently, the full texts of the remaining articles were assessed for eligibility. Ultimately, 13 full-text studies that met all inclusion criteria were included in this meta-analysis (Figure 1) [13,14,15,16,17,18,19,20,21,22,23,24,25]. The baseline characteristics of these 13 included studies are summarized in Table 1. All included trials compared clinical outcomes between TFL and Ho:YAG laser specifically for ureteroscopic lithotripsy, encompassing both semi-rigid URS and RIRS for upper urinary tract or kidney stones. The publication dates of the included studies ranged from 2020 to August 2025.

### 3.2. Quality Assessment and Publication Bias

The quality assessment of the included studies was performed according to the study design. Among the 13 included studies, six were RCTs and seven were non-randomized comparative studies. For the six RCTs, the risk of bias was evaluated using the RoB 2 tool (Table 2) [14,15,17,20,23,24].

Four studies (Martov et al., Ulvik et al., Chandramohan et al., and Gupta et al.) were assessed as having a low risk of bias across all domains [14,15,20,23]. The study by Haas et al. was rated as having some concerns in the domain of deviations from intended interventions [17]. Kaushik et al. was also rated as having some concerns in the randomization process domain due to the use of an alternation-based allocation method (odd/even numbers) [24]; however, the overall quality of the included RCTs was considered satisfactory.

For the seven non-randomized studies, the methodological quality was assessed using the MINORS index (Table 3) [13,16,18,19,21,22,25]. The scores ranged from 16 to 20 (out of 24), indicating a generally reliable methodological quality.

Publication bias was evaluated using funnel plots for the primary and secondary outcomes, including SFR, intraoperative complication rate, and postoperative complication rates (stratified by severity: Total, Clavien–Dindo I–II, and Clavien–Dindo III–IV). As shown in Figure S1, visual inspection of the funnel plots revealed a generally symmetrical distribution of the studies within the funnel limits. This symmetry suggests that there is no significant evidence of publication bias across the analyzed outcomes.

### 3.3. Heterogeneity Assessment and Model Selection

**Figure 2:**
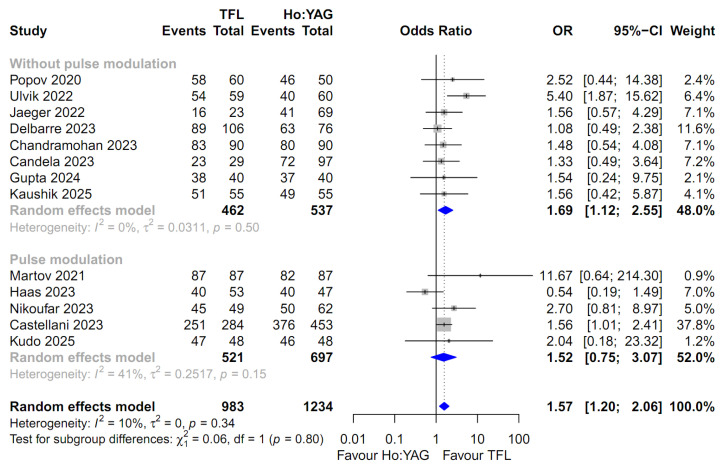
Forest plot comparing the stone-free rate (SFR) between Thulium Fiber Laser (TFL) and Holmium:YAG (Ho:YAG) laser. The analysis was stratified by the use of pulse modulation technology in the Ho:YAG group. CI, confidence interval; OR, odds ratio. The studies included in this forest plot are referenced as [13,14,15,16,17,18,19,20,21,22,23,24,25].

**Figure 3:**
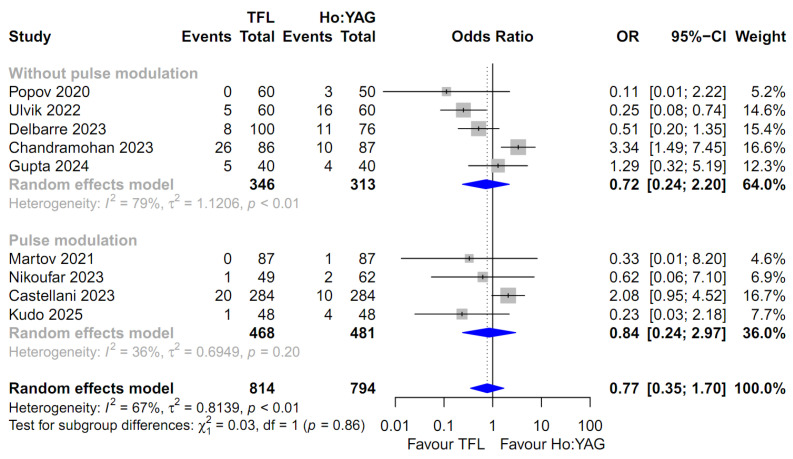
Forest plot comparing the intraoperative complication rate between Thulium Fiber Laser (TFL) and Holmium:YAG (Ho:YAG) laser. The analysis was stratified by the use of pulse modulation technology in the Ho:YAG group. CI, confidence interval; OR, odds ratio. The studies included in this forest plot are referenced as [13,14,15,18,19,20,22,23,25].

**Figure 4:**
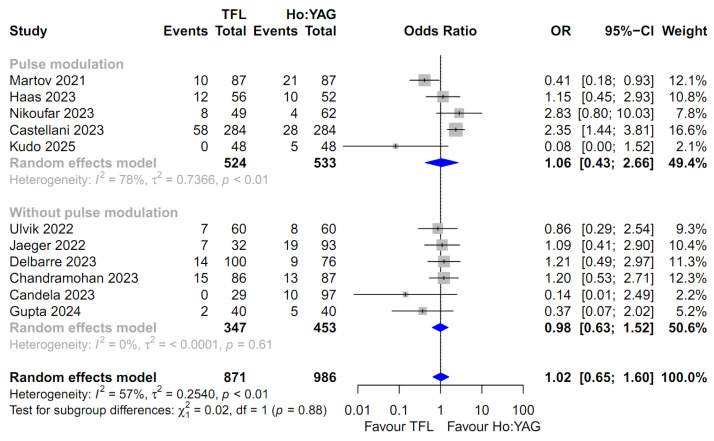
Forest plot comparing the total postoperative complication rate between Thulium Fiber Laser (TFL) and Holmium:YAG (Ho:YAG) laser. The analysis was stratified by the use of pulse modulation technology in the Ho:YAG group. CI, confidence interval; OR, odds ratio. The studies included in this forest plot are referenced as [14,15,16,17,18,19,20,21,22,23,25].

**Figure 5:**
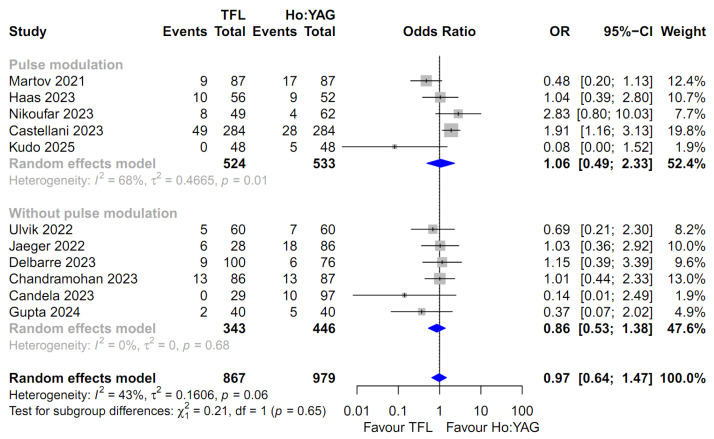
Forest plot comparing the minor postoperative complication rate (Clavien–Dindo grade I–II) between Thulium Fiber Laser (TFL) and Holmium:YAG (Ho:YAG) laser. The analysis was stratified by the use of pulse modulation technology in the Ho:YAG group. CI, confidence interval; OR, odds ratio.The studies included in this forest plot are referenced as [14,15,16,17,18,19,20,21,22,23,25].

**Figure 6:**
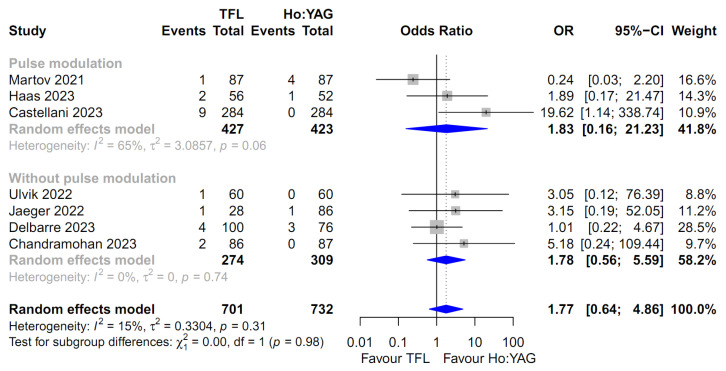
Forest plot comparing the severe postoperative complication rate (Clavien–Dindo grade III–IV) between Thulium Fiber Laser (TFL) and Holmium:YAG (Ho:YAG) laser. The analysis was stratified by the use of pulse modulation technology in the Ho:YAG group. CI, confidence interval; OR, odds ratio. The studies included in this forest plot are referenced as [14,15,16,17,18,20,22].

For the comparison of all outcomes, including the SFR, intraoperative complication rate, and postoperative complication rates (stratified by severity: Total, Clavien–Dindo I–II, and Clavien–Dindo III–IV), the random-effects model was uniformly employed. This approach was chosen to robustly account for the potential clinical diversity among the included studies and the observed heterogeneity in several subgroups (Figure 2, Figure 3, Figure 4, Figure 5 and Figure 6). Specifically, moderate-to-high heterogeneity was observed in the subgroup analyses for intraoperative (*I*^2^ = 67.2%) and total postoperative complications (*I*^2^ = 57.1%) (Figure 3 and Figure 4). Even for severe complications (Clavien–Dindo III–IV), where event rates were relatively low, the random-effects model was preferred to provide a conservative estimate of the pooled effect size, acknowledging the heterogeneity observed in the pulse modulation subgroup (*I*^2^ = 65%) (Figure 6).

### 3.4. Visual Assessment of Heterogeneity: L’Abbé Plots

L’Abbé plots were generated to visually assess the heterogeneity and the consistency of the treatment effects across the included studies (Figure S2). In these plots, the event rate in the Ho:YAG group is plotted on the *x*-axis against the event rate in the TFL group on the *y*-axis, with the diagonal line representing no treatment effect.

For the SFR, the data points are clustered near or slightly above the diagonal line, visually supporting the trend towards favorable outcomes for TFL, with relatively lower heterogeneity observed compared to complication rates.

Conversely, the L’Abbé plots for intraoperative and total postoperative complication rates show a considerable dispersion of study points around the diagonal line. This wide scattering visually confirms the substantial heterogeneity (*I*^2^ > 50%) identified in the statistical analysis, indicating that the relative safety profile of TFL versus Ho:YAG varies across different clinical settings and study designs.

For severe complications (Clavien–Dindo III–IV), the points are densely clustered near the origin (0, 0) due to the low incidence of events. However, the deviation of some points from the diagonal reflects the variability captured by the random-effects model, consistent with the moderate heterogeneity observed in the pulse modulation subgroup.

### 3.5. Further Assessment of Heterogeneity: Radial (Galbraith) Plots

Radial plots (Galbraith plots) were utilized to further investigate the sources of heterogeneity by plotting the standardized treatment effect (z-score) against the precision (reciprocal of the standard error) for each study (Figure S3). In these plots, studies that fall outside the 95% confidence interval (represented by the upper and lower limits around the central regression line) are considered potential outliers contributing to the observed heterogeneity.

For the intraoperative complication rate and total postoperative complication rate, the radial plots exhibited a distinct scatter pattern. Several studies were positioned outside the 95% confidence limits, visually confirming them as significant contributors to the high heterogeneity (*I*^2^ > 50%) detected in the forest plots. This suggests that variations in surgical techniques, laser settings, or definitions of complications across these specific studies may have driven the statistical inconsistency. In contrast, the radial plot for SFR showed that the majority of studies fell within or near the confidence limits, indicating a higher degree of consistency in the reported efficacy outcomes compared to safety outcomes. For severe complications, the plot revealed lower precision overall (points clustered to the left) due to the rarity of events, with a few deviations corresponding to the heterogeneity observed in the pulse modulation subgroup.

### 3.6. Stone-Free Rate

Thirteen studies involving 2217 patients were included in the analysis of the SFR [13,14,15,16,17,18,19,20,21,22,23,24,25]. The pooled analysis using a random-effects model demonstrated that the TFL group had a significantly higher SFR compared to the Ho:YAG group (OR 1.57; 95% CI: 1.20–2.06; *p* = 0.001, Figure 2). The overall heterogeneity among the included studies was low (*I*^2^ = 10.2%; *p* = 0.34).

Subgroup analysis was performed according to the use of pulse modulation technology in the Ho:YAG group. In the subgroup without pulse modulation (k = 8), the TFL group showed a statistically significant advantage in SFR compared to the Ho:YAG group (OR 1.69; 95% CI: 1.12–2.55; *I*^2^ = 0.0%). In the pulse modulation subgroup (k = 5), the SFR was higher in the TFL group (OR 1.52; 95% CI: 0.75–3.07; *I*^2^ = 41.2%), although this difference did not reach statistical significance. Notably, the test for subgroup differences indicated no significant disparity between the two subgroups (*p* = 0.80), suggesting that the beneficial trend of TFL remains consistent regardless of the pulse modulation capability of the comparator Ho:YAG laser.

To address the methodological heterogeneity regarding the definition of SFR across the included studies, we performed a sensitivity analysis stratifying the studies into a “strict SFR” group (defined as zero residual fragments; k = 5) and a “relaxed SFR” group (defined as residual fragments ≤ 2–4 mm; k = 8). The TFL group consistently demonstrated a higher odds ratio for SFR compared to the Ho:YAG group in both the strict (OR 1.85; 95% CI: 0.96–3.59) and relaxed (OR 1.54; 95% CI: 1.00–2.38) subgroups. Importantly, the test for subgroup differences indicated no significant disparity between the two definitions (*p* = 0.65). This confirms that the superior efficacy of TFL over Ho:YAG remains robust regardless of the stringency of the SFR definition (Figure S4).

Furthermore, an additional subgroup analysis was conducted to evaluate the impact of stone anatomical location and corresponding procedural type on laser efficacy. The studies were stratified into a “Ureteral Stones” group (managed primarily with semi-rigid URS; k = 5) and a “Renal or Mixed Stones” group (managed with RIRS; k = 8). In the renal or mixed stones subgroup, where the dusting capability of TFL is frequently utilized in restricted intrarenal spaces, TFL demonstrated a significantly higher SFR compared to Ho:YAG (OR 1.55; 95% CI: 1.01–2.39). A similar favorable trend for TFL was observed in the ureteral stones subgroup (OR 1.81; 95% CI: 0.94–3.51). The test for subgroup differences showed no significant variation between the two locations (*p* = 0.70), indicating that the clinical benefit of TFL is maintained across both ureteral and renal applications (Figure S5).”

Finally, to assess potential selection bias from pooling different study designs, we conducted a subgroup analysis comparing RCTs (k = 6) and non-randomized studies (k = 7). The positive effect of TFL on SFR was consistently observed in both the RCT (OR 1.80; 95% CI: 0.80–4.03) and non-RCT subgroups (OR 1.53; 95% CI: 1.12–2.11), with no significant difference between the two designs (*p* = 0.72). This confirms that the overall superiority of TFL is not disproportionately driven by non-randomized observational data (Figure S6).

### 3.7. Intraoperative Complication Rate

Nine studies involving 1608 patients were included in the analysis of the intraoperative complication rate [13,14,15,18,19,20,22,23,25]. The pooled analysis using a random-effects model showed no statistically significant difference in the intraoperative complication rate between the TFL and Ho:YAG groups (OR 0.77; 95% CI: 0.35–1.70; *p* = 0.52, Figure 3). Significant heterogeneity was observed among the included studies (*I*^2^ = 67.2%; *p* = 0.002).

Subgroup analysis based on pulse modulation was performed. In the subgroup without pulse modulation (k = 5), the TFL group showed a lower odds ratio, but the difference was not statistically significant (OR 0.72; 95% CI: 0.24–2.20). Considerable heterogeneity was noted in this subgroup (*I*^2^ = 78.9%). Similarly, in the subgroup with pulse modulation (k = 4), no significant difference was observed between the TFL and Ho:YAG groups (OR 0.84; 95% CI: 0.24–2.97), with moderate heterogeneity (*I*^2^ = 35.6%). The test for subgroup differences confirmed that the presence of pulse modulation did not significantly influence the comparative outcomes (*p* = 0.86).

### 3.8. Postoperative Complication Rate

Eleven studies involving 1857 patients were included in the analysis of the total postoperative complication rate [14,15,16,17,18,19,20,21,22,23,25]. The pooled analysis using a random-effects model showed no statistically significant difference between the TFL and Ho:YAG groups (OR 1.02; 95% CI: 0.65–1.60; *p* = 0.93, Figure 4). Significant heterogeneity was observed among the studies (*I*^2^ = 57.1%; *p* = 0.0096). Subgroup analysis demonstrated no significant difference between the groups regardless of pulse modulation (Test for subgroup differences: *p* = 0.88).

For minor postoperative complications (Clavien–Dindo grade I–II), eleven studies involving 1846 patients were analyzed [14,15,16,17,18,19,20,21,22,23,25]. The TFL group showed an odds ratio of 0.97 compared to the Ho:YAG group, which was not statistically significant (95% CI: 0.64–1.47; *p* = 0.90, Figure 5). Moderate heterogeneity was noted (*I*^2^ = 43.3%), and the subgroup analysis based on pulse modulation also revealed no significant differences (*p* = 0.65).

Regarding severe postoperative complications (Clavien–Dindo grade III–IV), seven studies involving 1433 patients were included [14,15,16,17,18,20,22]. The analysis indicated a higher odds ratio in the TFL group (OR 1.77; 95% CI: 0.64–4.86), but this difference was not statistically significant (*p* = 0.27, Figure 6). Heterogeneity was relatively low (*I*^2^ = 15.4%). Consistent with other outcomes, the subgroup analysis showed no significant interaction with pulse modulation (*p* = 0.98).

## 4. Discussion

Endoscopic lithotripsy has established itself as the mainstay of surgical treatment for upper urinary tract urolithiasis, driven by continuous advancements in endoscopic instrumentation. The evolution of flexible ureteroscopy necessitated the development of compatible lithotripsy devices. Consequently, the Ho:YAG laser became the dominant tool, offering superior safety over electrohydraulic lithotripsy and greater versatility than pneumatic lithotripsy [7,26,27]. However, as the complexity of endoscopic procedures increased, certain limitations of the Ho:YAG laser became apparent. These include the requirement for high-power outlets, low wall-plug efficiency (1–2%) necessitating bulky cooling systems, suboptimal wavelength matching for water absorption, and the “snowplow” effect or retropulsion of stones [4,28].

In contrast, the TFL offers several physical and technological advantages. TFL utilizes a standard power outlet and relies on air cooling due to its significantly higher energy efficiency (approximately 12%), allowing for a more compact device footprint [6,29]. Crucially, the wavelength of TFL (1940 nm) is much closer to the absorption peak of water (1910 nm) compared to that of the Ho:YAG laser (2120 nm). Consequently, the water absorption coefficient of TFL (129.2 cm^−1^) is approximately four times higher than that of Ho:YAG (31.8 cm^−1^) [29]. This physical profile translates into a four-fold-lower ablation threshold, enabling efficient stone dusting. Furthermore, TFL produces smaller vapor bubbles, which theoretically results in less retropulsion and collateral damage, potentially offering a better safety margin [7,30]. Additionally, TFL employs thinner silica fibers (50–150 μm), which occupy less space in the working channel, thereby improving irrigation flow and preserving the deflection capabilities of flexible ureteroscopes [4,31].

In this meta-analysis, we compared SFR, intraoperative complications, and postoperative complications between TFL and Ho:YAG laser. A key finding of our study is that TFL demonstrated a statistically significant superiority in SFR compared to the Ho:YAG laser overall. This superior efficacy is likely attributable to the TFL’s ability to ablate stones into finer dust (dusting mode) with reduced retropulsion, facilitating spontaneous passage of fragments. When stratified by pulse modulation, TFL showed a significantly higher SFR compared to Ho:YAG without pulse modulation. Although the difference did not reach statistical significance when compared to pulse-modulated Ho:YAG (e.g., MOSES technology), the favorable trend for TFL remained consistent. This suggests that while technological advancements in Ho:YAG (pulse modulation) have narrowed the efficiency gap by reducing retropulsion [5,32,33], TFL retains a high efficacy profile inherent to its continuous-wave-like properties and optimal wavelength.

Regarding safety, our analysis revealed no statistically significant differences in intraoperative or postoperative complication rates between TFL and Ho:YAG, regardless of pulse modulation. This contrasts with some preclinical expectations that the TFL’s shallow penetration depth (0.1–0.2 mm vs. 0.3–0.4 mm for Ho:YAG) might significantly reduce intraoperative injuries such as ureteral perforation or bleeding [30,34]. The lack of a statistical difference in clinical complications, despite the theoretical safety advantages of TFL, may be due to several factors. First, the high efficiency of TFL can lead to rapid temperature rise in the irrigation fluid if not managed with adequate flow, potentially offsetting its shallow penetration benefit. Second, substantial statistical heterogeneity (*I*^2^ > 50%) was observed in the complication analyses, particularly for intraoperative and total postoperative events. While our use of a random-effects model accounts for this mathematically, it is crucial to qualitatively address the underlying clinical sources of this dispersion. This clinical heterogeneity is likely multifactorial. First, there is considerable variation in the specific laser energy settings and irrigation protocols applied across the 13 included studies. TFL enables extremely high frequencies and unique dusting settings; however, aggressive energy applications without meticulously optimized fluid irrigation can lead to a rapid temperature rise, potentially increasing the risk of thermal mucosal injury. Second, discrepancies in surgeon experience play a pivotal role. Given that the Ho:YAG laser has been the surgical gold standard for decades, the relatively recent introduction of TFL means that some studies may reflect a learning curve effect, which can inherently impact operative times and complication rates. Third, differences in baseline stone burden and anatomical complexity (e.g., large or impacted stones requiring prolonged lithotripsy versus small, simple ureteral stones) significantly alter the baseline risk of adverse events. Finally, there is a lack of standardized criteria for defining and reporting minor intraoperative events—such as whether a minor mucosal abrasion is strictly classified as an intraoperative complication or considered a normal sequela of ureteroscopy. Collectively, these clinical variables largely explain the wide dispersion seen in our L’Abbé and radial plots. Nevertheless, our pooled results confirm that TFL maintains a comparable safety profile to the Ho:YAG laser overall, with no increase in severe complications (Clavien–Dindo III–IV).

Despite the inherent limitations of meta-analysis, our study possesses distinct strengths compared to previous systematic reviews. Unlike recent meta-analyses that included EAU Congress and AUA Annual Meeting abstracts [35,36,37,38,39] or combined data from PCNL [40,41], we strictly restricted our analysis to URS and RIRS using only full-text peer-reviewed articles. By excluding PCNL—which inherently differs from ureteroscopy in terms of invasiveness and stone clearance capabilities—and relying solely on peer-reviewed data, we minimized potential bias and heterogeneity related to surgical modality and data quality. Consequently, our findings derived specifically from URS and RIRS provide more homogeneous and clinically meaningful evidence for urologists performing endoscopic lithotripsy.

However, there are several limitations to this study. First, the analysis included 13 studies, of which only six were RCTs, while the others were non-randomized comparative studies. The inclusion of retrospective cohorts may introduce selection bias. However, our subgroup analysis demonstrated no significant difference in the effect sizes between RCTs and non-randomized studies, substantially mitigating this concern. Second, significant heterogeneity was observed, particularly in the analysis of complication rates, as visualized in the L’Abbé and radial plots. Third, the definition of “stone-free” and the imaging modalities used for assessment (CT vs. KUB/Ultrasound) varied across studies, which could influence the SFR outcomes. Fourth, our subgroup analysis comparing TFL with pulse-modulated Ho:YAG systems might be underpowered. Although we observed an Odds Ratio (OR) of 1.52 suggesting a potentially large clinical effect in favor of TFL, it did not reach statistical significance (*p* = 0.24), likely due to the limited number of studies (n = 5) in this specific subgroup. Finally, some recent studies could not be included due to overlapping patient populations or lack of extractable data for our specific endpoints [17,22,37]. Future large-scale, multicenter RCTs with standardized reporting criteria for complications and stone-free status are warranted to further validate these findings.

## 5. Conclusions

Ureteroscopic lithotripsy using the TFL demonstrates a superior SFR compared to the Ho:YAG laser overall. Specifically, TFL provides significantly better stone clearance than the Ho:YAG laser without pulse modulation and shows comparable, if not superior, efficacy to the Ho:YAG laser with pulse modulation technology. Regarding safety, TFL exhibits a favorable safety profile with intraoperative and postoperative complication rates comparable to those of the Ho:YAG laser. Therefore, TFL represents a highly effective and safe modality for the management of upper urinary tract stones. As clinical experience with TFL continues to accumulate, further optimization of surgical outcomes is anticipated. Future large-scale, randomized controlled trials are warranted to validate these findings and standardize optimal laser settings.

## Figures and Tables

**Table 1:** Characteristics of the studies included in the analysis.

Author Year	Country	Study Design	Inclusion Criteria	Type of Laser		No. Pts	Follow-Up	Definition of SFR	Quality Assessment (SIGN)
Popov et al. 2020 [13]	Russia	Cohort	Single ureter stone: 0.6~1 cm	TFL	Urolase SP, IPG Photonics	60	Not stated	No residual fragments	2+
				Ho: YAG laser (Without pulse modulation)	VersaPulse 100W, Lumenis	50			
Martov et al. 2021 [14]	Russia	RCT	Single ureter stone	TFL	Urolase SP, IPG Photonics	87	1 month NCCT	No residual fragments	1++
				Ho: YAG laser (Pulse modulation)	Pulse 120H, Lumenis	87			
Ulvik et al. 2022 [15]	Norway	RCT	Ureteral and/or renal stones ≥ 0.5 cm	TFL	Soltive Premium, Olympus	60	3 months NCCT	≤0.3 cm	1++
				Ho: YAG laser (Without pulse modulation)	Medilas H Solvo, Dornier MedTech	60			
Jaeger et al. 2022 [16]	USA	Pediatric Cohort	≤21 years old	TFL	SOLTIVE Premium SuperPulsed, Olympus	32	3 months US or KUB	No residual fragments	2+
			Ureteral and/or renal stones	Ho: YAG laser (Without pulse modulation)	Medilas H 20W, Medilas H Solvo 35 W	93			
Haas et al. 2023 [17]	USA	RCT	Non-staghorn stones < 2 cm	TFL	SOLTIVE Premium SuperPulsed, Olympus	56	3 months NCCT USG	≤0.3 cm	1+
				Ho: YAG laser (Pulse modulation)	P120H with MOSES Lumenis	52			
Delbarre et al. 2023 [18]	France	Non-Randomized controlled trial	Not stated	TFL	SOLTIVE Premium SuperPulsed, Olympus	100	3 months NCCT USG	≤0.3 cm	2+
				Ho: YAG laser (Without pulse modulation)	Medilas H20, Dornier MedTech	76			
Nikoufar et al. 2023 [19]	Canada	Cohort	Single renal stone	TFL	SOLTIVE Premium SuperPulsed, Olympus	49	1 months NCCT USG	≤0.4 cm	2+
				Ho:YAG laser (Pulse modulation)	P120H with MOSES Lumenis	62			
Chandramohan et al. 2023 [20]	India	RCT	Ureter (mid & distal) stone: 0.4~1.5 cm	TFL	Urolase SP, IPG Photonics	90	1 month NCCT KUB	≤0.2 cm	1+
				Ho:YAG laser (Without pulse modulation)	Litho 35 W, Quanta Systems	90			
Candela et al. 2023 [21]	International	Pediatric Cohort	Single renal stone < 2 cm	TFL	SOLTIVE Olympus Urolase SP IPG Photonics	29	3 months USG NCCT	≤0.2 cm	2+
				Ho:YAG laser (Without pulse modulation)	Lumenis 120W	97			
Castellani et al. 2023 [22]	International	Cohort	Renal stone	TFL	Not stated	284	3 months NCCT	≤0.2 cm	2+
				Ho:YAG laser (Pulse modulation)	P120H with MOSES Lumenis	284			
Gupta et al. 2024 [23]	India	RCT	Single ureter stone	TFL	Urolase SP IPG Photonics	40	1 month NCCT	No residual fragments	1+
				Ho:YAG laser (Without pulse modulation)	Not stated	40			
Kaushik et al. 2025 [24]	India	RCT	Ureteric stone	TFL	Not stated	55	1 month KUB USG	No residual fragment	1+
				Ho:YAG laser (Without pulse modulation)	Not stated	55			
Kudo et al. 2025 [25]	Japan	Cohort	Ureteral and/or renal stone	TFL	FiberLase IPG	48	1 month NCCT	≤0.2 cm	2+
				Ho:YAG laser (Pulse modulation)	P120H with MOSES Lumenis	48			

Notes: Abbreviations: TFL, Thulium Fiber Laser; Ho:YAG, Holmium:Yttrium-Aluminum-Garnet; RCT, Randomized Controlled Trial; NCCT, Non-Contrast Computed Tomography; KUB, Kidney, Ureter, and Bladder X-ray; USG, Ultrasonography; SFR, Stone-Free Rate; SIGN, Scottish Intercollegiate Guidelines Network. Evidence quality was assessed using the SIGN grading system: 1++, High-quality meta-analyses, systematic reviews of RCTs, or RCTs with a very low risk of bias. 1+, Well-conducted meta-analyses, systematic reviews, or RCTs with a low risk of bias. 2++, High-quality systematic reviews of case–control or cohort studies; or high-quality case–control or cohort studies with a very low risk of confounding or bias and a high probability that the relationship is causal. 2+, Well-conducted case–control or cohort studies with a low risk of confounding or bias and a moderate probability that the relationship is causal. Manufacturer locations for the laser systems are as follows: IPG Photonics (Oxford, MA, USA), Lumenis (Yokneam, Israel), Olympus (Tokyo, Japan), Dornier MedTech (Wessling, Germany), and Quanta System (Samarate, Italy).

**Table 2:** Risk of Bias for Included RCTs (RoB 2).

Study ID	Randomization (D1)	Deviations (D2)	Missing Data (D3)	Measurement (D4)	Reporting (D5)	Overall
Martov et al. 2021 [14]	Low	Low	Low	Low	Low	Low
Ulvik et al. 2022 [15]	Low	Low	Low	Low	Low	Low
Haas et al. 2023 [17]	Low	Some concerns	Low	Low	Low	Some concerns
Chandramohan et al. 2023 [20]	Low	Low	Low	Low	Low	Low
Gupta et al. 2024 [23]	Low	Low	Low	Low	Low	Low
Kaushik et al. 2025 [24]	Some concerns	Low	Low	Low	Low	Some concerns

Note: Risk of bias was assessed using the Cochrane Risk of Bias 2 (RoB 2) tool for randomized controlled trials. Abbreviations: D1, randomization process; D2, deviations from intended interventions; D3, missing outcome data; D4, measurement of the outcome; D5, selection of the reported result.

**Table 3:** Methodological Quality of Non-randomized Studies (MINORS Scale).

Study	1	2	3	4	5	6	7	8	9	10	11	12	Total
Popov et al. 2020 [13]	2	2	1	2	1	2	2	0	2	2	1	0	17
Jaeger et al. 2022 [16]	2	2	0	2	0	2	1	0	2	2	1	2	16
Delbarre et al. 2023 [18]	2	2	2	2	1	2	2	1	2	2	2	0	20
Nikoufar et al. 2023 [19]	2	2	0	2	0	2	2	0	2	2	1	2	17
Candela et al. 2023 [21]	2	2	0	2	0	2	2	0	2	2	2	2	18
Castellani et al. 2023 [22]	2	2	0	2	0	2	2	0	2	2	2	2	18
Kudo et al. 2025 [25]	2	2	0	2	0	2	2	0	2	2	1	2	17

Notes: The methodological quality of non-randomized studies was assessed using the Methodological Index for Non-randomized Studies (MINORS). For comparative studies, each of the following 12 items was scored as 0 (not reported), 1 (reported but inadequate), or 2 (reported and adequate), with a maximum total score of 24: 1. A clearly stated aim. 2. Inclusion of consecutive patients. 3. Prospective collection of data. 4. Endpoints appropriate to the aim of the study. 5. Unbiased assessment of the study endpoint. 6. Follow-up period appropriate to the aim of the study. 7. Loss to follow up less than 5%. 8. Prospective calculation of the study size. 9. An adequate control group. 10. Contemporary groups 11. Baseline equivalence of groups. 12. Adequate statistical analyses.

## Data Availability

The data presented in this study are available in the article.

## Supplementary Materials

The following supporting information can be downloaded at: https://www.mdpi.com/article/10.3390/medicina62040644/s1, Table S1: PRISMA Checklist [42], Figure S1: Funnel plots for evaluating publication bias, Figure S2: L’Abbé plots for visual assessment of heterogeneity, Figure S3: Radial (Galbraith) plots for further investigation of heterogeneity, Figure S4: Forest plot of the sensitivity analysis for the stone-free rate (SFR) based on the stringency of the SFR definition, Figure S5: Forest plot of the subgroup analysis for the stone-free rate (SFR) based on stone anatomical location, Figure S6: Forest plot of the subgroup analysis for the stone-free rate (SFR) based on study design.

## Abbreviations

AUA	American Urological Association
CI	Confidence interval
EAU	European Association of Urology
Ho:YAG	Holmium:yttrium-aluminum-garnet
KUB	Kidney, ureter, and bladder x-ray
MD	Mean difference
MeSH	Medical Subject Headings
MINORS	Methodological Index for Non-Randomized Studies
NCCT	Non-contrast computed tomography
OR	Odds ratio
PCNL	Percutaneous nephrolithotomy
PRISMA	Preferred Reporting Items for Systematic Reviews and Meta-Analyses
RCT	Randomized controlled trial
RIRS	Retrograde intrarenal surgery
RoB	Risk of Bias
SFR	Stone-free rate
SIGN	Scottish Intercollegiate Guidelines Network
SWL	Shock wave lithotripsy
TFL	Thulium fiber laser
URS	Ureteroscopy
USG	Ultrasonography
