# Supplementary material for: Thulium Fiber Laser Versus Holmium Laser for Ureteroscopic Lithotripsy: A Systematic Review and Meta-Analysis
Source: Medicina (Kaunas). 2026 Mar 28;62(4):644. doi: 10.3390/medicina62040644 (PMC13117054; doi:10.3390/medicina62040644)
Supplement: Supplementary file 1 [file medicina-62-00644-s001.zip › Figure S3.pdf]

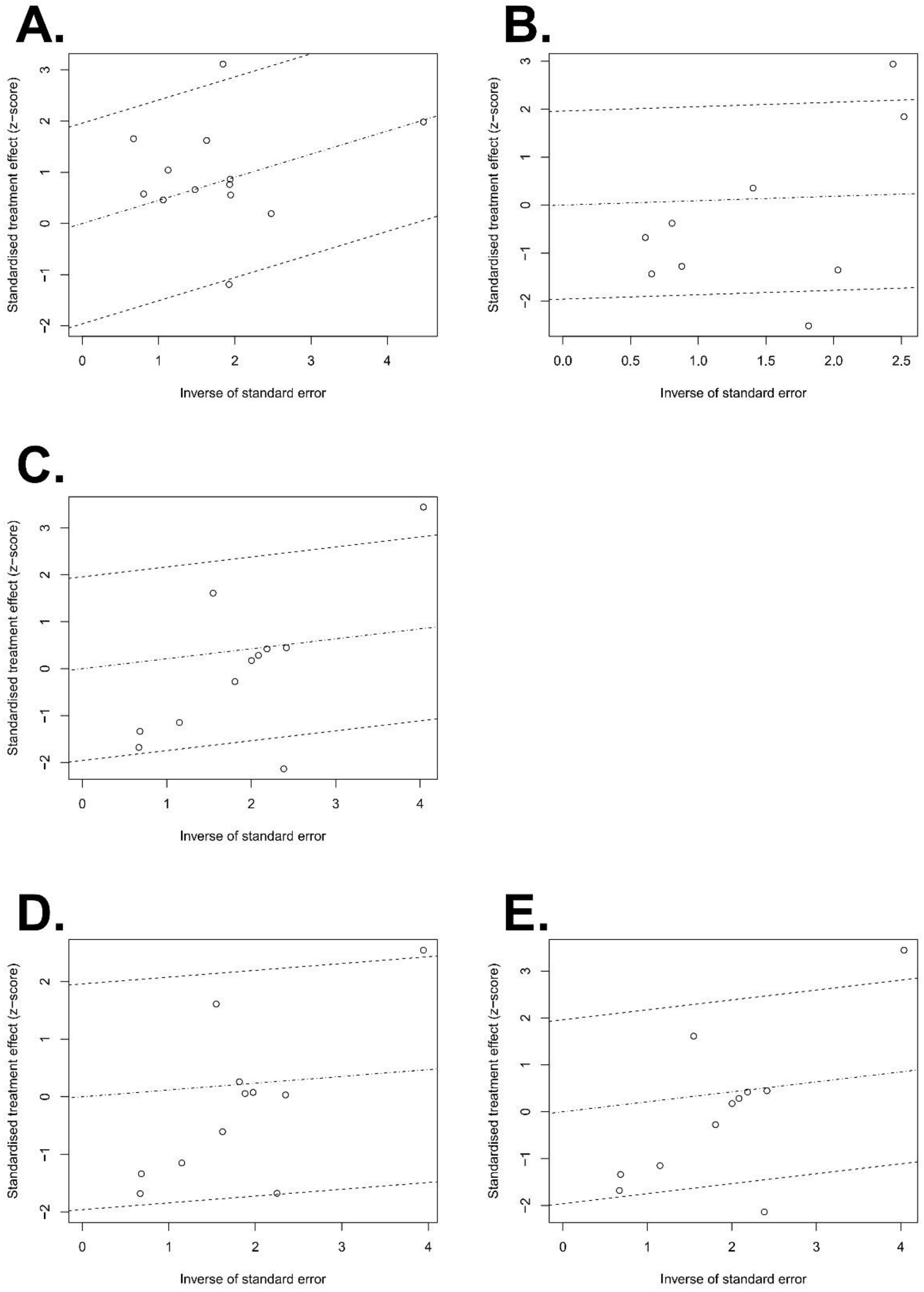

Figure S3. Radial (Galbraith) plots for further investigation of heterogeneity. (A) Stone-free rate (SFR). (B) Intraoperative complication rate. (C) Total postoperative complication rate. (D) Minor postoperative complication rate. (E) Severe postoperative complication rate. Studies outside the 95% confidence interval (lines) are potential sources of heterogeneity.
