# Supplementary material for: Thulium Fiber Laser Versus Holmium Laser for Ureteroscopic Lithotripsy: A Systematic Review and Meta-Analysis
Source: Medicina (Kaunas). 2026 Mar 28;62(4):644. doi: 10.3390/medicina62040644 (PMC13117054; doi:10.3390/medicina62040644)
Supplement: Supplementary file 1 [file medicina-62-00644-s001.zip › Figure S4.pdf]

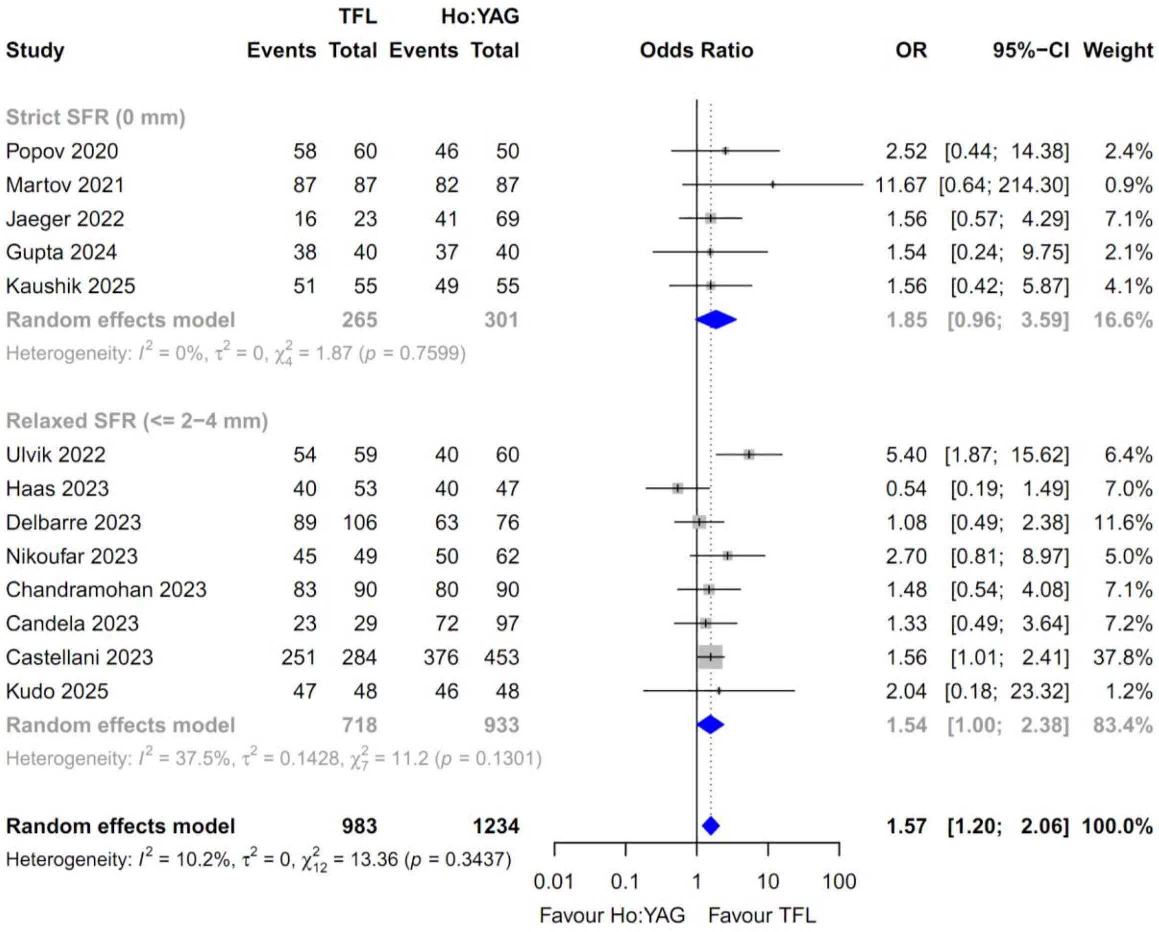

Figure S4. Forest plot of the sensitivity analysis for the stone-free rate (SFR) comparing Thulium Fiber Laser (TFL) and Holmium:YAG (Ho:YAG) laser. The analysis was stratified by the stringency of the SFR definition across the included studies: a “strict SFR” subgroup (defined as zero residual fragments) and a “relaxed SFR” subgroup (defined as residual fragments ≤ 2–4 mm). CI, confidence interval; OR, odds ratio; SFR, stone-free rate.
