# Supplementary material for: Thulium Fiber Laser Versus Holmium Laser for Ureteroscopic Lithotripsy: A Systematic Review and Meta-Analysis
Source: Medicina (Kaunas). 2026 Mar 28;62(4):644. doi: 10.3390/medicina62040644 (PMC13117054; doi:10.3390/medicina62040644)
Supplement: Supplementary file 1 [file medicina-62-00644-s001.zip › Figure S5.pdf]

Study	TFL Events	TFL Total	Ho:YAG Events	Ho:YAG Total	OR	95%−CI	Weight
**Ureteral Stones (URS)**							
Popov 2020	58	60	46	50	2.52	[0.44; 14.38]	2.4%
Martov 2021	87	87	82	87	11.67	[0.64; 214.30]	0.9%
Chandramohan 2023	83	90	80	90	1.48	[0.54; 4.08]	7.1%
Gupta 2024	38	40	37	40	1.54	[0.24; 9.75]	2.1%
Kaushik 2025	51	55	49	55	1.56	[0.42; 5.87]	4.1%
**Random effects model**		**332**		**322**	**1.81**	**[0.94; 3.51]**	**16.6%**
Heterogeneity: $I^2 = 0\%$, $\tau^2 = 0$, $\chi^2_4 = 1.94$ ($p = 0.7467$)							
**Renal or Mixed Stones (RIRS)**							
Ulvik 2022	54	59	40	60	5.40	[1.87; 15.62]	6.4%
Jaeger 2022	16	23	41	69	1.56	[0.57; 4.29]	7.1%
Haas 2023	40	53	40	47	0.54	[0.19; 1.49]	7.0%
Delbarre 2023	89	106	63	76	1.08	[0.49; 2.38]	11.6%
Nikoufar 2023	45	49	50	62	2.70	[0.81; 8.97]	5.0%
Candela 2023	23	29	72	97	1.33	[0.49; 3.64]	7.2%
Castellani 2023	251	284	376	453	1.56	[1.01; 2.41]	37.8%
Kudo 2025	47	48	46	48	2.04	[0.18; 23.32]	1.2%
**Random effects model**		**651**		**912**	**1.55**	**[1.01; 2.39]**	**83.4%**
Heterogeneity: $I^2 = 37.5\%$, $\tau^2 = 0.1430$, $\chi^2_7 = 11.2$ ($p = 0.1302$)							
**Random effects model**		**983**		**1234**	**1.57**	**[1.20; 2.06]**	**100.0%**
Heterogeneity: $I^2 = 10.2\%$, $\tau^2 = 0$, $\chi^2_{12} = 13.36$ ($p = 0.3437$)							

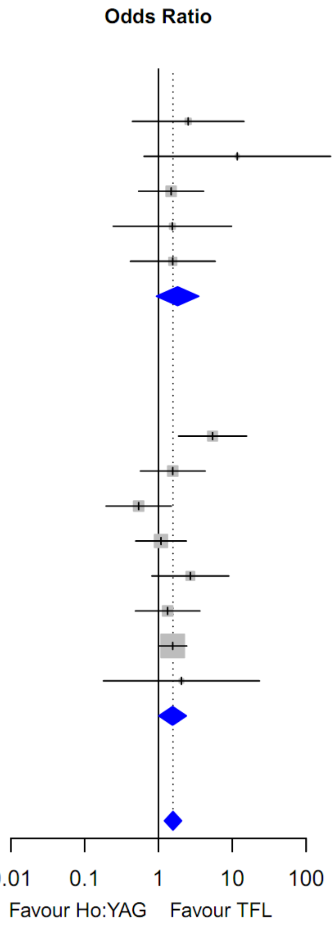

Figure S5. Forest plot of the subgroup analysis for the stone-free rate (SFR) comparing Thulium Fiber Laser (TFL) and Holmium:YAG (Ho:YAG) laser based on stone anatomical location. The included studies were stratified into a “Ureteral Stones” subgroup (managed primarily with semi-rigid ureteroscopy [URS]) and a “Renal or Mixed Stones” subgroup (managed with retrograde intrarenal surgery [RIRS]). CI, confidence interval; OR, odds ratio.
