# Supplementary material for: Thulium Fiber Laser Versus Holmium Laser for Ureteroscopic Lithotripsy: A Systematic Review and Meta-Analysis
Source: Medicina (Kaunas). 2026 Mar 28;62(4):644. doi: 10.3390/medicina62040644 (PMC13117054; doi:10.3390/medicina62040644)
Supplement: Supplementary file 1 [file medicina-62-00644-s001.zip › Figure S6.pdf]

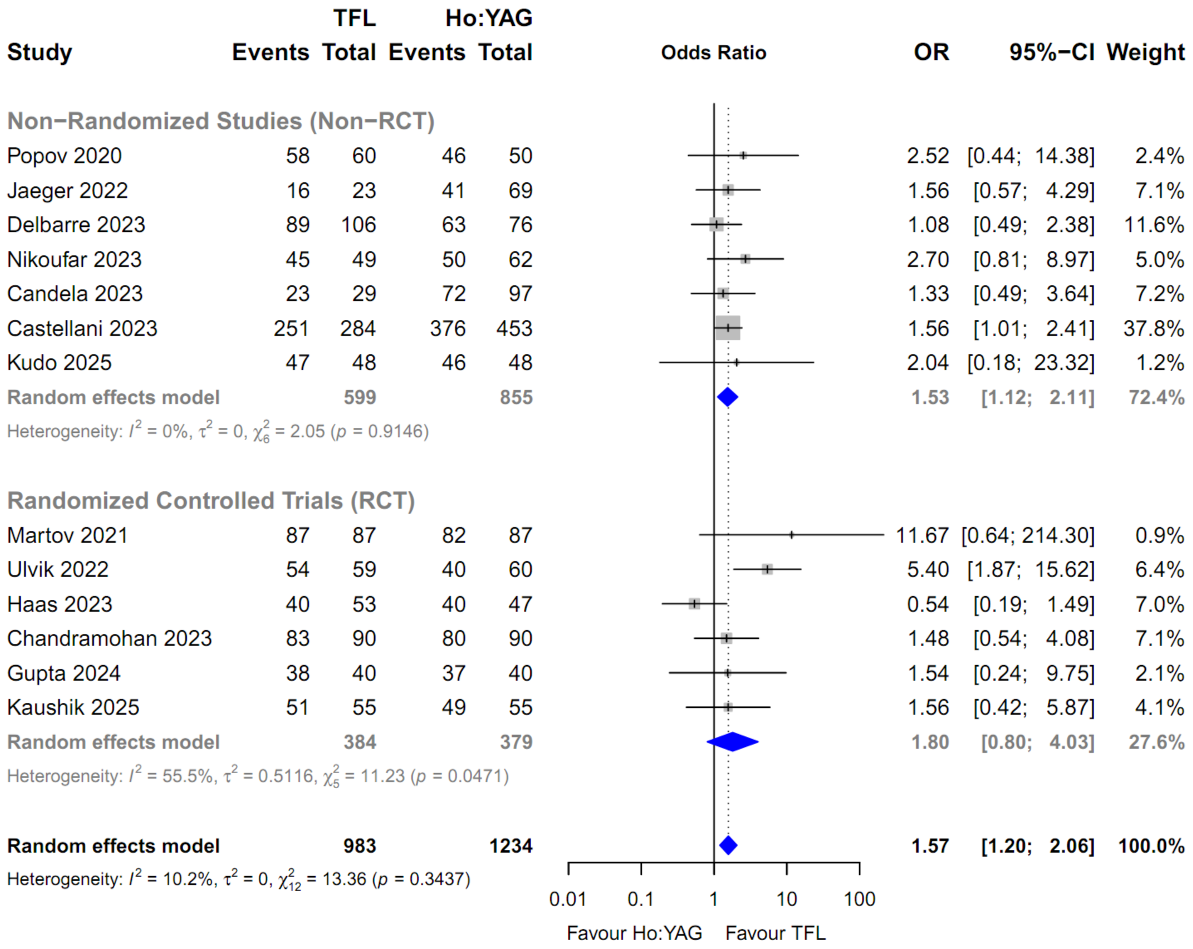

Figure S6. Forest plot of the subgroup analysis for the stone-free rate (SFR) comparing Thulium Fiber Laser (TFL) and Holmium:YAG (Ho:YAG) laser based on study design. The included studies were stratified into a "Randomized Controlled Trials (RCT)" subgroup and a "Non-Randomized Studies (Non-RCT)" subgroup to assess potential selection bias. CI, confidence interval; OR, odds ratio.
